# Uterus transplantation and beyond

**DOI:** 10.1007/s10856-017-5872-0

**Published:** 2017-03-29

**Authors:** Mats Brännström

**Affiliations:** Department of Obstetrics and Gynecology, Sahlgrenska Academy, University of Gothenburg, Göteborg, Sweden and Stockholm IVF, Stockholm, Sweden

## Abstract

Uterus transplantation is today the only available treatment for absolute uterine factor infertility which is caused by either congenital/surgical uterine absence or that a present uterus is non-functioning. Structured animal-based research, from rodents to nonhuman primates, was the scientific basis for a successful introduction of uterus transplantation as a clinical procedure. The patient groups for uterus transplantation, the preclinical research and data from the published human cases will be covered herein. During recent years the concept of bioengineering of organs and tissues has emerged. Creation of a bioengineered uterus is in the initial research state, with experiments performed in rodents. The research that has been performed to create a bioengineered uterus will be summarized. In conclusion, uterus transplantation is now a clinical experimental procedure for treatment of uterine factor infertility. In parallel to the establishment of this combined assisted reproduction technique and transplantation procedure as a routine clinical procedure, we predict that uterus bioengineering will develop further towards introduction within the human setting, but that this process will take several years.

## Introduction

Absolute uterine factor infertility (AUFI) has for many years been regarded as untreatable. The first, but unsuccessful uterus transplantation (UTx) case, was performed in 2000 with an organ from a live donor [1] and this was followed by the second attempt in 2011 with the uterus from a deceased donor [2]. Both these attempts were done without any research preparations and do not comply with the IDEAL (Innovation, Development, Exploration, Assessment, Long-term follow up) concept for introduction of surgical innovations [3] and the international ethics guidelines for UTx [4].

Our research group initiated animal-based UTx-research in 1999 and has by a step-by-step approach subjected most aspects of an UTx procedure to experiments in rodents, domestic species and non-human primates [5] prior to our clinical trial, with the world´s first live birth after UTx demonstrated in 2014 [6].

In recent years, we have also launched a project with the aim to create a bioengineered uterus for future UTx. The advantage of this approach would be to circumvent organ shortage, avoid surgery in a live donor situation and that the transplanted patient can be without immunosuppression, since the organ will be created by the recipients own stem cells. We are in the initial phase of this research project and predict that this will, in line with the UTx project, take at least a decade from the laboratory to the clinical setting.

In this article the potential patient groups for UTx are described. The systematic animal-based research efforts, that formed the foundation for human UTx, are also explained. Human UTx is in its infancy and at the very early experimental stage. The initial 11 human UTx attempts, that also have been scientifically published, and the outcomes of these are described in detail in this review. These attempts were performed during the period between 2000 and 2013. Noteworthy is that the same number of new attempts have taken place and been reported in the media during the last 12 months and also that the early surgical success, with remaining grafts after 1 month, is considerably lower than of the cohort from 2000 until 2013. The last section of this review covers a possible, future extension UTx, which is creation of a bioengineered uterus. This research line is in its early stages and it is predicted that it will take at least a decade until this spreads to the clinical setting.

### Potential patient groups for UTx and bioengineered uterus

The patients that are in need for a uterus are those that have the absolute uterine factor infertility (AUFI) condition. This may be caused by either a lack of the uterus or that the existing uterus is non-functional in terms of carrying a pregnancy beyond the gestational length that would allow for a live birth. The AUFI causes are listed in Table 1.

**Table 1:** Causes of uterine factor infertility that may be treatable by uterine transplantation

No uterus
Congenital uterine absence (Müllerian agenesis/Mayer-Rokitansky-Küster-Hauser (MRKH)-syndrome)
Hysterectomy
Cervical/uterine malignancy
Leiomyoma
Obstetric bleeding
Atony
Malplacentation (placenta accreta/percreta)
Uterine rupture
Uterus present
Leiomyoma
Adenomyosis
Multiple miscarriage/implantation failure
Radiation damage
Uterine malformation
Partly unicornuate uterus
Partly bicornuate uterus
Hypoplastic uterus
Cervical incompetence with multiple miscarriages
Post multiple conisation procedures
Post trachelectomy procedure
Intrauterine adhesions not treatable by hysteroscopic resection

Concerning bioengineering of only a partial uterus or uterine tissue, additional indications would be those patients that have been operated with resection of normal uterine tissue, and with a large risk for miscarriage or uterine rupture secondary to this. The reasons for uterine resections may be leiomyoma, adenomyosis or invasive malplacentation (placenta accreta/percreta).

Based on calculations in the UK [7], 1:500 women of fertile age are estimated to be infertile because of uterine factor. In North America, that would correspond to more than 50,000 women.

### Animal-based UTx research

Modern attempts in animal-based UTx research stems from the early 2000s [8, 9]. Key findings of this research are summarized below.

#### Rodents

**Fig. 1:**
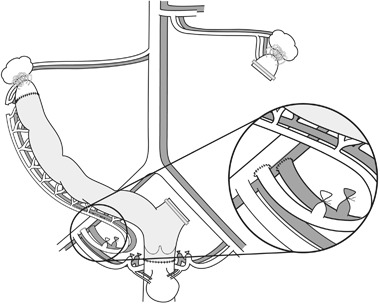
Schematic drawing of the rat uterus transplantation model. The graft is with the left uterine horn excised to enable only unilateral vascular anastomoses, which are end-to-end on the common iliac artery and vein on the right side. The uterus is anastomosed to the vagina and to a minor, cranial segment of the native uterus, to allow for spontaneous conception with uninterrupted tubal-uterine junction

The first ever offspring after UTx was demonstrated in 2003 [10]. That was in the mouse model, although in a syngeneic setting. Offspring exhibited normal growth trajectory and fertility. Subsequent experiments in this species demonstrated tolerability to 24 h of cold ischemia [11]. The rejection process after allogeneic UTx in the mouse was characterized regarding morphology, blood flow [12] and leukocyte infiltration [13]. Later, a rat UTx model (Fig. 1) was developed [14], that allowed for normal conception [15]. In the allogeneic rat UTx model, tacrolimus was found superior to cyclosporine to prevent rejection [16–18]. Pregnancies were demonstrated after allogeneic UTx in the rat with tacrolimus treatment [19] and normal growth of offspring was seen [20].

#### Domestic species

Both the pig and sheep have been used in UTx-research, with the major advantage over the rodents being the larger sizes of the organs and uterine vasculature. Initial attempts in the sheep were with autologous UTx and anastomosis to the external iliac vessels [21, 22] also demonstrating live offspring [23]. In the allogeneic sheep UTx-model, initially long-term uterine survival [24] was seen and this was followed by demonstration of a live birth [25]. Triple immunosuppression, with tacrolimus, mycophenolate mofetil (MMF) and prednisolone was also tested in the sheep UTx model [26].

The pig UTx model was initially developed as autologous transplantation, examining reperfusion events [27] and vascular patency [7]. Later, long-term survival after allo-UTx in the pig was seen after initial treatment with tacrolimus for 2 weeks, which was followed by cyclosporine [28].

#### Nonhuman primates

**Fig. 2:**
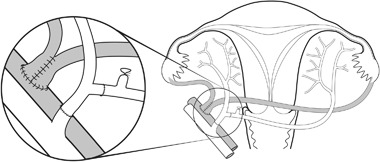
Schematic drawing of the autologous uterus transplantation model in the baboon. The ovaries and oviducts are included in the graft. At back-table preparation ovarian veins are fused side-to-side and the internal iliacs of the graft are reconstructed to allow for one arterial inlet. Anastomoses are then end-to-side on the external iliacs

The baboon and cynomolgus macaque have been used in preclinical UTx research. In our initial UTx study in the baboon with autologous transplantation, restored menstruation was seen only in 20% of animals [29]. After modification of organ flushing and anastomosis surgery (Fig. 2), a 3-fold higher success rate was demonstrated [30]. In allogeneic UTx in the baboon, with transplantation from live donors, recovery surgery lasted for 3–4 h and donor survival was 100% [31]. An induction protocol with antithymocyte globulin, followed by tacrolimus, MMF and corticosteroids was effective to suppress rejection [31] and a rejection-grading system, based on histology of cervical biopsies, was developed in the baboon [31, 32]. Survival of the allogeneically transplanted baboon uteri was seen for over 12 months [32].

Initial UTx studies with autologous transplantation in the smaller cynomolgus macaque, using bilateral anastomosis of the uterine artery and the deep uterine vein to the external iliacs demonstrated resumed menstruation [33]. It was also shown that the complete macaque uterus is adequately perfused with use of only unilateral anastomosis [34]. Importantly, the first UTx pregnancy and live offspring, albeit after autologous UTx, in a primate species was shown in the cynomolgus macaque [35]. Moreover, a surgical procedure, using the ovarian vein for outflow, demonstrated adequate perfusion after UTx [36] and it was concluded that this technique could potentially simplify live donor UTx-surgery. Recently, allogeneic UTx in the macaque, with triple antirejection therapy (tacrolimus, MMF and methylprednisolone) was performed and resumed menstruation was seen around 3 months after surgery [37].

### Preclinical human UTx research

There exist a small number of preclinical human UTx studies. We assessed the tolerability to cold ischemia of human myometrial tissue-pieces from hysterectomy specimens and found that the morphology, on the electron microscopy level, was well preserved after 6 h of cold ischemia and spontaneous, as well as prostaglandin-augmented, contractions were present [38].

Two studies investigated uterine recovery from brain-dead donors. In the initial study, uterine donation with a research purpose was only accepted by the relatives of the brain-dead women of the New York City region in 6% of cases [39]. The other study was performed in France, almost a decade later [40], when the concept of human UTx was known to the public after initial success of human UTx [6]. In that study, with a much higher acceptance rate, organ retrieval procedure was performed in seven female multi-organ donors. The uterus was removed together with the internal iliac vessels, parametria, and vaginal fornices. They found no major morphologic changes after 24 h of cold ischemia and the vessels could be preserved in all except one case of unilateral vein loss.

In a live donor UTx procedure, it is more difficult to acquire long vascular pedicles. We explored this issue in a study of patients undergoing radical hysterectomy surgery for cervical cancer and with added separate dissection of uterine arteries and veins. The accomplished lengths of the uterine arteries and veins were around 6 cm, which would enable bilateral end-to-side anastomosis to the external iliac vessels [41].

### Human UTx-cases

The first human UTx attempt was performed in Saudi Arabia, in April 2000 [1]. The premenopausal donor was 46-year-old and the recipient was a 26-year-old woman, that had undergone emergency peripartum hysterectomy 6 years before. The donor hysterectomy was with short uterine vessels and extensive back-table surgery was performed, by anastomosing great saphenous venous grafts on all vessels. A perioperative ureteric laceration was repaired during surgery. The donor uterus, with vessel extensions, were anastomosed into the recipient end-to-side to the external iliacs. Cyclosporine, corticosteroids and azathioprine were used as immunosuppression. Spontaneous menstruations were not seen. On day 99, the recipient presented with pelvic heaviness and vaginal discharge. Uterine blood flow was found to be absent and an infarcted uterus was removed. Albeit the early graft failure, this human UTx cases advanced the field considerably by demonstrating the feasibility of the surgical procedures in a live donor and the recipient. However, it was clear that animal-based research was necessary before any new clinical attempts.

The second human UTx, with a uterus from a deceased 22-year-old donor, was performed in Turkey in 2011 [2]. Similarly to the first human UTx case in year 2000, also this was without any research preparations. The recipient was a 21-year old patient with uterine agenesis. Uterus recovery, with the uterus as the prioritized organ, took 2 h and transplantation, including bilateral end-to-side anastomosis of the common iliacs of the graft to the external iliac vessels, lasted 6 h. Daily thymoglobulin was given for 10 days, and this was followed by combined tacrolimus, prednisolone and MMF. The uterus remains today, almost 6 years after UTx, still in the recipient. Despite multiple embryo transfer attempts over 4 years, no pregnancy with a viable heart activity has occurred [42]. The reason for the pregnancy failure is not clear but it may well be related to uterine-specific factors rather than embryo-specific factors, since the recipient is of young age with the associated higher oocyte quality and that the young donor uterus had not demonstrated its ability to carry a pregnancy before.

In Sweden, we performed nine live-donor UTx procedures in early 2013 [43]. Eight recipients had congenital uterine agenesis and one had undergone a radical hysterectomy because of cervical cancer. Prior to surgery the recipients went through IVF treatments to cryopreserve embryos. The donors were mothers in five cases, one sister, one maternal aunt, one mother-in-law and one close friend. Donors, recipients and partners of recipients were, prior to surgery, extensively screened concerning medical and psychological factors [44].

The donor surgery [43] was through a midline incision and the uterus was gently dissected avoiding damage to the ureters and the adjacent uterine vessels. Bilateral vascular pedicles were dissected to include the internal iliac arteries, distal to the branching of the gluteal artery, as well as the major uterine veins down to, and including parts of the internal iliac veins. The donor surgery lasted 10.5–13 h and with a hospital stay of 6 days for all donors. In one patient, a ureteric-vaginal fistula was diagnosed after 2 weeks and this was later repaired. She has no sequela.

**Fig. 3:**
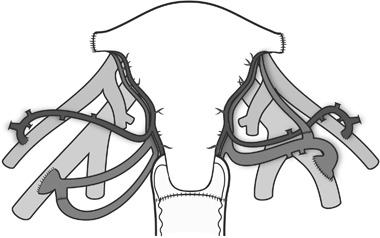
Schematic drawing of vascular connections in human uterus transplantation from live donors. The anterior branches of the internal iliac arteries and segments of the internal iliac veins of the donor are anastomosed end-to-side to the external iliac vessels. On the left side the upper uterine vein, which anatomically converges with the ovarian vein, has been anastomosed to the internal iliac vein of the graft to increase the venous outflow. On the right side one of the ovarian veins were transected before organ retrieval to enable removal of the graft with an intact ureter. The transected vein was reanastomosed end-to-end at back-table preparation

After flushing and cooling of the uterus ex vivo, it was transplanted into the recipient by bilateral end-to-side anastomoses to the external iliacs (Fig. 3), vaginal anastomosis and uterine fixation to several pelvic ligaments. Induction immunosuppression was given with two doses of thymoglobulin plus methylprednisolone and maintenance therapy was with tacrolimus and MMF. After 8 months, MMF was either discontinued or replaced by azathioprine, depending on the frequency of rejection episodes. During the initial months, hysterectomy had to be performed in two patients. One uterus was removed after 3 days because of bilateral thrombosis of the uterine vessels. The other uterus was removed 3.5 months after surgery because of intrauterine infection, that developed into an abscess and septicemia [43] These two graft failures occurred in two out of the three uteri that were from donors above age 60 at transplantation and also with the lowest uterine blood flow just after transplantation. These factors may be related to graft failures.

The seven remaining uteri showed spontaneous and regular menstruations from 1–2 months after UTx and uterine artery blood flow was within normal ranges [45]. Subclinical, mild rejection episodes were diagnosed on protocol cervical biopsies in five out of seven women [45]. The psychological outcome during the first post-transplantation year of recipients and partners was an overall optimism but with minor anxiety around graft survival around 3 months post UTx [46].

Embryo transfers with single embryos were performed from 12–14 months after UTx. Two women became pregnant at their first embryo transfer [6, 47] attempts and the first live birth after UTx took place in September 4, 2014 [6]. In that pregnancy, in a patient with uterine and unilateral renal agenesis, preeclampsia developed at gestational week 31 + 5. Delivery was by c-section the following day. The second UTx-baby in the world was from a mother-to-daughter transplantation and delivery of a healthy baby was by c-section in week 34 + 4 [47]. In our trial [43] there have been four more births of healthy babies and two more pregnancies are ongoing with expected deliveries in the first half of 2017 (our unpublished observations). These are the only successful human UTx attempts so far.

### The future of human UTx

Uterus transplantation is still an experimental procedure and should stay at this phase until enough scientific data has accumulated to ensure that UTx is a reasonable safe and effective procedure. An international registry to follow donors, recipients and children born has been formed as part of the activities of the International Society of Uterus Transplantation (ISUTx). Data from that registry will be important to monitor the safety of the procedure, concerning long-term effects of the participants and children born after UTx. Several clinical trials of UTx are now launched in North-America, Europe and Asia. Based on the favourable outcome so far from the first UTx trial [43], it is predicted that UTx will be in clinical routine at several centers worldwide within 5 years.

### Bioengineered uterus

There exist several disadvantages with classical allogeneic UTx and these may be partly overcome by the new principle of tissue engineering to create a bioengineered uterus. In traditional UTx, there is a risk of tissue rejection and immunosuppressant-related side effects such as increased risks of hypertension, diabetes, nephrotoxicity and accelerated arteriosclerosis [48].

There is also the issue of organ availability, which however may be a minor problem concerning UTx. In the case of UTx, the pool of living donors is predicted to be large, considering that the uterus is without any important physiological function after childbearing. Thus, altruistic living donation from related and non-related donors will most likely be common, in particular when the surgery has developed further to considerably reduced surgery times and after the introduction of minimal invasive laparoscopic techniques for organ harvesting. Moreover, there would also be the pool of deceased donors, but it should be emphasized that the concept of deceased donor UTx has not yet proven to be successful.

**Fig. 4:**
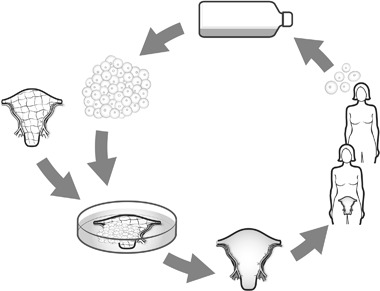
General principle for construction of a bioengineered uterus. Stem cells of the future recipient are expanded in vitro. A uterine scaffold (synthetic scaffold or a biologically derived scaffold) is then cellularized by stem cells in vitro in a bioreactor. The bioengineered organ is then transplanted into the recipient, who will not need any immunosuppressive medication and can keep the organ for many years with no side-effects

Development of a bioengineered uterus is based on the principle that the uterus is mostly created from the recipients own stem cells (Fig. 4), that grow on a synthetic scaffold or a biologically derived scaffold. This concept will circumvent the problem of exposing the recipient to immunosuppression drugs and will also possible decrease organ shortage. Novel and promising concepts for functional organ- or tissue replacement have emerged within the fields of regenerative medicine and tissue engineering on a wide range of organs and tissues [49].

Tissue engineering involves several steps, from the development of a scaffold to tissue reconstruction using various cell sources. A uterine scaffold could be either synthetic or biologically derived to provide support for structure, cell proliferation and differentiation.

Biological scaffolds mimic the native organ mechanically, geometrically and biologically to a greater extent than synthetic scaffolds. These biological scaffolds are typically obtained by decellularization, a process to remove the immune activating cells and to leave a framework of three-dimensional extra cellular matrix (ECM). The ECM provides organ-specific tissue architecture with preserved vascular conduits [50]. It contains important molecules, mainly type I collagen, glycosaminoglycans, fibronectin, laminin, elastin and possibly some growth factors with tissue specific composition. These molecules provide signals for cell aggregation, adhesion, migration, proliferation and differentiation [51].

Decellularization is achieved by exposing the organ to a combination of either detergents, to ionic- or non-ionic solutions, to physical forces (such as freeze-thawing and high-/low pressure), or to various enzymatic treatments. These methods are non-selective and will also partly damage ECM elements. Thus, a balance is needed between an aggressive enough decellularization for removal of immunogenic DNA and intracellular components while maintaining the ECM microenvironment intact, including patent conduits for the vasculature [50].

Concerning the uterus, clean scaffolds of collagen/matrigel [52] and silk/collagen [53] have been tested. In one study in the rat, collagen membranes were loaded with bone marrow derived mesenchymal stem cells (BM-MSC) and then transplanted to areas of partial full thickness uterine excisions [54] and it was shown that the BM-MSCs would facilitate regeneration of the tissue and ingrowth of blood vessels.

Different decellularization protocols have been tested on rat uterine tissue in a small number of studies.

In one study [55], decelluarization of uterine tissue pieces (5 × 15 mm) with either sodium dodecyl sulphate (SDS), Triton-X100 or high hydrostatic pressure (HHP) were compared. The Triton-X100 protocol achieved less decellularization than the other two protocols (SDS and HHP). A benefit of the HHP product, as compared to after SDS, may be that with a similar collagen content, the mechanical strength was in the same range at that of the native uterus.

The other two studies concerning rat uterus decellularization utilized whole organs and with administration of the agents through the vasculature. One study perfused the rat uterus via the aorta with SDS in distilled water for 72 h. Absence of nuceli and cytoplasmic proteins were found, and importantly with preservation of ECM proteins such as laminin and collagen type 1 [56]. There existed signs of a preserved vascular bed, since perfusion with red dye showed flow from the large vessels to the capillaries of the uterine scaffold. In our initial study we compared three different decellularization protocols [57]. Two protocols were based on a combination of Triton-X100 and dimethyl sulfoxide (DMSO), where one protocol was buffered by phosphate buffered saline (PBS) for the duration of the procedure, while the other utilized non-buffered deionized H_2_O. Our third protocol was based on the ionic detergent sodium deoxycholate (SDC) in deionized H_2_O. Decelluarization was then achieved by perfusion of the agents through the vasculature. Immunohistochemistry of the decellularized tissue showed that all three protocols effectively removed the immune reactive elements of major histocompatibility (MHC) class I and II. Mechanical tests, transmission electron microspopy and proteomic analysis revealed some differences and it was decided that all three protocols would be compared in our subsequent study [58] on recelluarization and retransplantation.

Before progressing further on whole-uterus bioengineering applications, or move to larger animal models, we should try to establish which scaffold design is the most suitable for the cellular reconstruction and the successive in vivo applications. The uterine scaffold may be implanted directly in vivo to recruit repopulating endogenous cells from the host, or as more commonly applied, cells can be integrated into the scaffold prior to transplantation in a process generally termed recellularization. One major challenge is to find an appropriate cell source for recellularization. For whole-organ engineering, an ideal cell type is one that can proliferate and give rise to all cell types necessary for the particular organ to be regenerated, including the parenchyma, stroma, the vasculature and all other supporting structures. To date, embryonic- and mesenchymal stem cells are the most prevalent cell types used for recellularization [59]. However, it is likely a mix of cell types will be required for a successful reconstruction and future work will also focus on what sequence the different cell types should be applied in.

A successful recellularization also requires optimal cell delivery methods and culturing conditions. The two main methods for cell delivery are either by perfusion of cells through the vasculature, or by repeated injections of cells into the scaffold. Perfusion would be the choice in order to reach the vasculature, whereas injections of cells target the parenchyma and stroma more directly. Thus, a combination of the two has been the approach by most groups in this research area. The culturing conditions also matters on the recellularization efficiency, and one of the advantages with using decellularized biological tissues is that the vascular conduits are preserved. This route is therefore commonly used to cannulate and connect to various custom made or commercially available perfusion bioreactors.

To our knowledge, there exist only four reports on recellularization of decellularized uterine scaffolds. Two studies implanted decellularized tissue into the normal uterine compartment to allow for full recellularization in vivo. Uterine tissue pieces of rats that had been decellularized with either SDS or HHP were transplanted as patches into the native uterus and evaluation was after an in vivo period of 30 days [55]. Regeneration of all compartments were seen and blood vessels were present within the tissue. Proliferation was seen mostly in the luminal epithelium and pregnancy occurred in the horns that contained the recellularized tissue, although it was not demonstrated that placentation occurred over these specific areas. In a follow up paper in the mouse, the same research-group showed that in animals that had undergone excision of the ovaries recellularization was hormone independent, but that STAT3 is important in the recellularization process since cell proliferation was suppressed in uteri of *Stat3* conditional knockout mice [60].

The other two studies [56, 58], performed in the rat, used the approach of recellularization in vitro and with tissue that had been decellularized as complete uterus with perfusion through the vasculature. Recellularization was performed on the whole construct [56] or on segments of these decellularized uteri [58] with cell mixtures of mesenchymal stem cells (MSCs) and primary uterine cells in both studies. Miyazaki and Maruyama [56] could show partial distribution of uterine cells with initial formation of endometrium after 3 days in the bioreactor but with a progressive atrophy after that time. There were no attempts to reimplant these in vitro recellularized whole constructs, but the study demonstrated adequate recellularization of empty decellularized tissue segments that were reimplanted into the uterus. We compared three different uterine scaffolds that were decelluarized by perfusion in vitro and then recellularized by a 1:150 mixture of primary uterine cells and green fluorescent protein (GFP) labeled MSCs [58]. The recellularization was in vitro and on small patches that was cultured for 3 days after injection of cells into the scaffolds. The partly recellularized patches were then transplanted for further in vivo experiments. After in vitro recellularization there was only a limited cell distribution, but after transplantation the scaffold was repopulated by host uterine cells that eventually replaced the GFP-labeled cells. Pregnancy occurred in uterine horns with patches but placentation was not seen over the patch areas.

## Conclusion

Uterus transplantation is now at its initial experimental stage. The extensive animal-research that preceded a structured and scientific introduction of human UTx, with an initial prospective observational study, was most likely important for that several births now have been reported from the nine women that entered the trial. It is essential that new human attempts of UTx are done within the framework of clinical trials in order to accumulate data to further optimize the procedure in relation to safety and efficiency. In this regard the creation of a global registry to follow donors, recipients and children born after UTx is central.

It may well be that classical UTx procedure, with transplantation from live or deceased donors, will only stay as the predominant infertility treatment for AUFI women for one or two decades, since creation of a bioengineered uterus may enter the clinical arena in the future. However, the research on this bioengineered uterus solution has only been ongoing for a few years and there are likely several obstacles that have to be solved before that can be tested in a human setting.
